# Serum Dioxin Concentrations and Bone Density and Structure in the Seveso Women’s Health Study

**DOI:** 10.1289/ehp.1306788

**Published:** 2013-11-15

**Authors:** Brenda Eskenazi, Marcella Warner, Marcella Sirtori, Thomas Fuerst, Stephen A. Rauch, Paolo Brambilla, Paolo Mocarelli, Alessandro Rubinacci

**Affiliations:** 1 Center for Environmental Research and Children’s Health (CERCH), School of Public Health, University of California at Berkeley, Berkeley, California, USA; 2 Bone Metabolic Unit, San Raffaele Scientific Institute, Milano, Italy; 3 Synarc Inc., San Francisco, California, USA; 4 Department of Laboratory Medicine, University of Milano-Bicocca, School of Medicine, Hospital of Desio, Desio-Milano, Italy

## Abstract

Background: 2,3,7,8-Tetrachlorodibenzo-*p*-dioxin (TCDD), a widespread environmental contaminant, is a known endocrine disruptor. In animal studies, TCDD exposure impairs bone metabolism and increases fragility. To our knowledge, no epidemiologic studies have examined this association.

Objectives: On 10 July 1976, a chemical explosion in Seveso, Italy, resulted in the highest known residential exposure to TCDD. In 1996, we initiated the Seveso Women’s Health Study, a retrospective cohort study of the health of the women. In 2008, we followed up the cohort. Here, we evaluated the association between TCDD exposure and bone structure and geometry in adulthood, and considered whether timing of TCDD exposure before achievement of peak bone mass (assumed to occur 2 years after onset of menarche) modified the association.

Methods: Individual TCDD concentration was measured in archived serum collected soon after the explosion. In 2008, 350 women who were < 20 years old in 1976 underwent a dual-energy X-ray absorptiometry (DXA) bone scan. Bone mineral density was measured at the lumbar spine and hip, and hip geometry was extracted from hip DXA scans using the hip structural analysis method.

Results: Among premenopausal women, TCDD serum levels were associated with some indexes indicating better bone structure in women exposed before peak bone mass (*n* = 219), with stronger associations in those exposed before 5 years of age (*n* = 46). In contrast, among postmenopausal women, TCDD levels were associated with evidence of better bone structure in women exposed after peak bone mass (*n* = 48) than in other women (*n* = 18).

Conclusions: Our current results do not support the hypothesis that postnatal TCDD exposure adversely affects adult bone health. Continued follow-up of women who were youngest at exposure is warranted. Future studies should also focus on those exposed *in utero*.

Citation: Eskenazi B, Warner M, Sirtori M, Fuerst T, Rauch SA, Brambilla P, Mocarelli P, Rubinacci A. 2014. Serum dioxin concentrations and bone density and structure in the Seveso Women’s Health Study. Environ Health Perspect 122:51–57; http://dx.doi.org/10.1289/ehp.1306788

## Introduction

2,3,7,8-Tetrachlorodibenzo-*p*-dioxin (TCDD or dioxin), a lipophilic compound with a half-life in humans of 7–9 years (Needham et al. 1994; Pirkle et al. 1989), is a common contaminant in industrialized areas. In animal studies, TCDD exposure disrupts a wide range of endocrine-mediated functions (Birnbaum 1994, 1995; International Agency for Research on Cancer 1997), mostly via effects on the aryl hydrocarbon receptor (AhR) (Hankinson 1995; Okey et al. 1994; Okino and Whitlock 2000). Bone metabolism is a hormonally dependent process, and estrogen signaling is important for normal bone development and homeostasis (Sambrook and Cooper 2006). It has been proposed that TCDD may disrupt bone metabolism directly via the AhR, which is expressed in both osteoblasts (responsible for bone formation) and osteoclasts (responsible for bone resorption) (Ilvesaro et al. 2005) or via cross-talk between the AhR and estrogen receptor (ER) α/β signaling pathways (Kietz et al. 2004; Nilsson et al. 2001; Ohtake et al. 2003).

Bone mass increases throughout childhood with a marked acceleration during puberty, when estradiol serves to increase bone density and suppress bone remodeling, resulting in an increase in cortical thickness (Wang et al. 2004). The greatest increase in female bone mass occurs from 12 to 15 years of age, then slows by 18 years (Davies et al. 2005; Theintz et al. 1992). Although peak bone mass is primarily determined by genetics, hormone levels and environmental factors may also contribute (Davies et al. 2005).

In rats, exposure to TCDD and dioxin-like polychlorinated biphenyls (PCBs) has been reported to increase bone fragility (Jamsa et al. 2001; Lind et al. 2000; Miettinen et al. 2006) and decrease bone growth, modeling, and mechanical strength (Jamsa et al. 2001). It has been suggested that higher exposure to dioxin-like compounds could account, at least in part, for the observed increase in osteoporosis and osteoporotic fractures in the industrialized world (Cooper et al. 2011; Ström et al. 2011). After a review of the evidence, Davies et al. (2005) proposed that exposure during the critical window for bone mass growth in childhood and adolescence could lower peak bone mass and increase risk for osteoporotic fractures after menopause. To our knowledge, no epidemiologic study has examined the relationship of early-life exposure to TCDD and bone health.

On 10 July 1976, an explosion at a chemical plant in Seveso, Italy, released up to 30 kg of TCDD over the surrounding 18-km^2^ area, which was divided into exposure zones (A, B, R, non-ABR) based on surface soil TCDD measurements (Di Domenico et al. 1980). In 1996, we initiated the Seveso Women’s Health Study (SWHS), a historical cohort study examining the effect of TCDD exposure on women’s health. In the present study, we investigated the relationship of TCDD measured in blood collected soon after the explosion (Mocarelli et al. 1990) and bone mineral density (BMD) and bone structure > 30 years later. We examined whether exposure to TCDD is associated with adult bone mass and whether this association is modified by exposure before the attainment of peak bone mass.

## Methods

*Study population*. Details of the study design are presented elsewhere (Eskenazi et al. 2000). Eligible women were newborn to 40 years of age in 1976, resided in zones A or B (the most highly contaminated zones), and had a blood sample collected soon after the explosion that was of adequate volume for analysis of TCDD. Of 1,271 eligible women, 981 were alive in 1996 and willing to participate. In 2008, we followed up the SWHS cohort; 426 women who were < 20 years of age in 1976 who were not currently pregnant were invited to undergo a BMD scan of the hip and spine, and 350 participated. These women were younger and more highly exposed than the full cohort (Eskenazi et al. 2004). For the current analysis, we excluded women with Turner’s syndrome (*n* = 2) or who reported currently taking corticosteroids (*n* = 8). Hip and spine BMD scans were not analyzable for one subject each, lowering the sample size to 339. This study was approved by the institutional review boards of the participating institutions, and written informed consent was obtained from all women before participation.

*Procedure*. At the 2008 follow-up, women underwent a fasting blood draw, anthropometric measurements, and a structured personal interview administered by a nurse. In addition, women < 20 years of age underwent a dual-energy X-ray absorptiometry (DXA) bone scan.

The interview collected information about demographic and lifestyle characteristics, reproductive, personal, and family medical history, and food intake using the European Prospective Investigation into Cancer and Nutrition–Italy food frequency questionnaire (Pisani et al. 1997).

Participants were asked a series of questions about their menstrual cycles to ascertain menopause status. Women were coded as perimenopausal if they identified that category, or if they identified as menopausal, but their last menstrual period was < 1 year before the study. Women were coded as menopausal if they self-identified as such and if their last menstrual period was ≥ 1 year before. Women who had had hysterectomies or other medical procedures that induced menopause were coded as menopausal.

*Laboratory analysis*. We measured TCDD in archived sera by high-resolution gas chromatography/high-resolution mass spectrometry methods (Patterson et al. 1987). Values are reported on a lipid-weight basis in parts per trillion (ppt) (Akins et al. 1989). For the current analysis sample, we measured TCDD in sera collected in 1976–1977 for 314 women (92.4%); in 1978–1981 for 21 women (6.2%); and in 1996–1997 for the 5 women (1.5%) who had insufficient volume in earlier samples [see Eskenazi et al. (2000, 2004) for details on sample selection]. For nondetectable values (*n* = 28), a serum TCDD level of one-half the detection limit was assigned (Hornung and Reed 1990). The median serum sample weight was 0.65 g, median limit of detection was 18.8 ppt, lipid-adjusted, and the analytic coefficient of variation was 15% (5% within day and 10% between days). For women with detectable post-1977 TCDD measurements of > 10 ppt, TCDD levels were back-extrapolated to 1976 using the first-order kinetic model (Pirkle et al. 1989) for women who were > 16 years of age in 1976 (*n* = 4) or the Filser model (Kreuzer et al. 1997) otherwise (*n* = 17). For two women whose post-1977 TCDD values were detectable but ≤ 10 ppt, the measured value was used.

*BMD assessment*. To avoid scanning pregnant women, we scheduled scans during or soon after menses (Centers for Disease Control and Prevention 1989). All bone density scans were performed by the same physician (M.S.), who was blinded to the woman’s zone of residence and TCDD level. BMD (grams per centimeter squared) measurements were made at the lumbar spine (L1–L4) and left proximal femur (neck, trochanter, intertrochanter, and total hip) using DXA (Discovery A; Hologic Inc., Waltham, MA, USA) at the Bone Metabolism Unit of the Scientific Institute of San Raffaele in Milan, Italy. Precision error was < 0.5%, and the *in vivo* coefficient of variation was 1.0% for the measurement sites. All DXA scans were reviewed (by T.F.) to ensure that the regions of interest were properly defined. Quality assurance of the DXA scanner was performed before use, and long-term instrument stability was assessed by daily calibration using a phantom.

*Hip structural analysis (HSA)*. Hip structural geometry was extracted from hip DXA scans using the HSA method (Beck 2002). The HSA program uses mineral content and dimensional data from conventional DXA images of the hip to measure the structural dimensions of bone cross-sections corresponding to three thin regions traversing the proximal femur: the narrow neck region across the narrowest point of the femoral neck, the intertrochanteric site across the bisector of the neck and shaft axes, and the femoral shaft region located at a distance 1.5 times the width of the femoral neck distal to the intersection of the neck and shaft axes. At these three regions, we estimated bone size [outer diameter (defined as subperiosteal width), cross-sectional area, cortical thickness, and buckling ratio] and bone strength [cross-sectional moment of inertia (referred to as bending stress) and section modulus (referred to as bending strength)]. Higher numbers indicate greater bone strength except for the buckling ratio, which is an index of susceptibility to local cortical buckling under compressive loads and is derived from the ratio of periosteal width to cortical thickness. Lower buckling ratio indicates greater cortical stability, and therefore, greater bone strength.

*Data analysis*. Serum TCDD levels were log_10_-transformed. We explored the shape of the relationship between TCDD and BMD and HSA using generalized additive models (splines with 3 degrees of freedom) as well as categorical exposure and covariate variables; there was no evidence of nonlinearity (data not shown). For premenopausal women (the majority of our sample), the International Society for Clinical Densitometry recommends the use of age-standardized *z*-scores (Lewiecki et al. 2004). We defined low BMD as a *z*-score ≤ –1 (Kanis et al. 1997; Lewiecki et al. 2004). We used NHANES (National Health and Nutrition Examination Survey) reference values for Caucasian women to calculate *z*-scores (Looker et al. 1998); these values have been found to be similar for the 1,622 women 20–79 years of age in the Densitometric Italian Normative Study, a multicenter study that aims to establish reference values for bone densitometry with DXA in the Italian population (Pedrazzoni et al. 2003). BMD measures at the spine and hip were modeled as continuous *z*-scores and binary outcomes (high or low, as defined above). HSA outcomes were treated as continuous variables. We used linear regression to examine the relation of serum TCDD with BMD *z*-score and geometric properties at the three proximal femur regions for HSA outcomes, and logistic regression to examine the relation of serum TCDD with low BMD (*z*-score ≤ –1 vs. > –1).

We considered the following as possible confounders: ages at explosion, menarche, and bone density scan; menarche status at explosion; education; parity; breastfeeding history; physical activity; youth athletic training; current and lowest adult body mass index (BMI); smoking and alcohol status; vitamin D, calcium, and milk intake; family or personal history of osteoporosis, breast cancer, other cancers, thyroid problems, or infertility; use of oral contraceptives, other hormones (including fertility drugs, hormone pills, creams, injections, and skin patches, and non-oral hormonal contraception), or past steroid medications (subjects currently taking steroid medications were excluded from the analysis); and hysterectomy. Covariate status is current unless otherwise specified. Covariates were included in the final multivariate models if they changed the coefficient for log_10_TCDD by > 10%.

Because BMD undergoes extensive changes after menopause (Sowers et al. 1992), we tested for effect modification by menopause status at the time of DXA scan (premenopause vs. perimenopause/menopause). In addition, to examine whether associations differed based on timing of TCDD exposure, we tested for effect modification by exposure occurring before or after peak bone mass [defined for each participant as 2 years after her self-reported age at menarche (Theintz et al. 1992)] and by exposure before or after 5 years of age (Alaluusua et al. 2004). We also assessed statistical interactions between log_10_TCDD and current thyroid-stimulating hormone and BMI, examining each variable in continuous and categorical/binary forms; no interactions were significant for any of the outcomes (with significant interactions defined as *p* < 0.2 for a cross-product interaction term).

In sensitivity analyses, we repeated the final models excluding the following: women with a history of breast cancer (because treatments could affect bone loss) (*n* = 5), thyroid problems (*n* = 52), or osteoporosis (*n* = 3), or current hormone replacement therapy (*n* = 5); no exclusions substantially altered the results (data not shown).

## Results

Of the 340 women, 274 (80.6%) were premenopausal, 21 (6.2%) were perimenopausal, and 45 (13.2%) were menopausal. Women averaged (± SD) 10.7 ± 5.0 years at explosion and 43.3 ± 5.0 years at follow-up. As shown in Table 1, most women were premenarche at explosion (55%), and most were parous at follow-up (81%). About half the women currently or previously smoked. Average BMI for the group was 25.3 ± 4.9 kg/m^2^, with only 2% underweight (BMI < 18.5) but 44% overweight or obese (BMI ≥ 25). About half of the women reported a family history of osteoporosis, and 15% reported having a thyroid problem.

**Table 1:** Select characteristics of participants who were < 20 years old in 1976 and underwent DXA stratified by menopause status, Seveso Women’s Health Study, Italy, 2008–2009 [*n* (%)].

Characteristic	All women	Premenopause	Perimenopause/menopause
Total	340 (100.0)	274 (80.6)	66 (19.4)
Age at explosion (years)
0–4	47 (13.8)	46 (16.8)	1 (1.5)
5–9	79 (23.2)	75 (27.4)	4 (6.1)
10–14	129 (37.9)	114 (41.6)	15 (22.7)
15–19	85 (25.0)	39 (14.2)	46 (69.7)
Menarche status at explosion
Premenarche	187 (55.0)	176 (64.2)	11 (16.7)
Postmenarche	153 (45.0)	98 (35.8)	55 (83.3)
Age at bone density scan (years)
30–34	15 (4.4)	14 (5.1)	1 (1.5)
35–39	77 (22.7)	75 (27.4)	2 (3.0)
40–44	101 (29.7)	97 (35.4)	4 (6.1)
45–52	147 (43.2)	88 (32.1)	59 (89.4)
Education
≤ Required	148 (43.5)	111 (40.5)	37 (56.1)
High school	173 (50.9)	147 (53.7)	26 (39.4)
> High school	19 (5.6)	16 (5.8)	3 (4.6)
Parity
0	64 (18.8)	59 (21.5)	5 (7.6)
1	91 (26.8)	75 (27.4)	16 (24.2)
≥ 2	185 (54.4)	140 (51.1)	45 (68.2)
Smoking status
Never	186 (54.7)	147 (53.7)	39 (59.1)
Former	76 (22.4)	63 (23.0)	13 (19.7)
Current	78 (22.9)	64 (23.4)	14 (21.2)
Alcohol status
Never	239 (70.3)	198 (72.3)	41 (62.1)
Former	10 (2.9)	7 (2.6)	3 (4.6)
Current	91 (26.8)	69 (25.2)	22 (33.3)
BMI (kg/m^2^)
< 18.5	7 (2.1)	6 (2.2)	1 (1.5)
18.5–24.9	183 (53.8)	154 (56.2)	29 (43.9)
25–29.9	106 (31.2)	87 (31.8)	19 (28.8)
≥ 30	44 (12.9)	27 (9.9)	17 (25.8)
Height (cm)
≤ 152	36 (10.6)	27 (9.9)	9 (13.6)
152.5–160	135 (39.7)	103 (37.6)	32 (48.5)
160.5–167	118 (34.7)	100 (36.5)	18 (27.3)
> 167	51 (15.0)	44 (16.1)	7 (10.6)
Family history of osteoporosis
No	169 (49.7)	139 (50.7)	30 (45.5)
Yes	171 (50.3)	135 (49.3)	36 (54.5)
Oral contraceptive (OC) use
Never	70 (20.6)	57 (20.8)	13 (19.7)
Former	214 (62.9)	164 (59.9)	50 (75.8)
Current	56 (16.5)	53 (19.3)	3 (4.6)
Non-OC hormone use
Never	280 (82.4)	229 (83.6)	51 (77.3)
Former^*a*^	54 (15.9)	42 (15.3)	12 (18.2)
Current^*b*^	6 (1.8)	3 (1.1)	3 (4.6)
Steroid medication use^*c*^
Never	297 (87.4)	242 (88.3)	55 (83.3)
Former	43 (12.7)	32 (11.7)	11 (16.7)
Thyroid problems (any)^*d*^
No	288 (84.7)	233 (85.0)	55 (83.3)
Yes	52 (15.3)	41 (15.0)	11 (16.7)
^***a***^Former hormones include vaginal ring, pill, cream, patch, injection, or fertility medications. ^***b***^Current hormones include vaginal ring, pill, or patch. ^***c***^Steroid medications include oral prednisone and cortisone. ^***d***^Thyroid problems include hypothyroid (14), hyperthyroid (8), Graves’ disease (2), Hashimoto’s disease (4), nodules (16), thyroid cancer (1), thyroiditis (4), benign tumor/cyst (1), goiter (1), other (1).			

The distribution of serum TCDD levels for all women, and by menopause status, is shown in Supplemental Material, Table S1. The median (interquartile range) serum TCDD concentration soon after the explosion was 73.2 (33.1–193.0) ppt for all women and 78.9 (40.9–209.0) ppt and 43.1 (21.9–129.0) ppt for premenopause and perimenopause/menopause groups, respectively. As presented in Table 2, women averaged slightly lower hip BMD than expected for their age (mean ± SD *z*-score for total hip = –0.11 ± 0.98) but slightly higher spine bone mass (0.08 ± 1.13). Approximately 18% of women had low BMD either of the spine or hip, defined as a *z*-score ≤ –1.0. The percent of low BMD was higher in the perimenopause/menopause group (23% for spine and 27% for total hip) than in the premenopause group (17% for both). Table 2 presents a summary of the HSA outcomes by menopause status. The perimenopause/menopause group had a significantly higher buckling ratio at all three hip locations (narrow neck, femoral shaft, intertrochanter). The perimenopause/menopause group also had lower bone mineral density (grams per centimeter squared) than the premenopause group, as well as a larger outer diameter in the hip narrow neck and femoral shaft.

**Table 2:** Summary of measures (mean ± SD) of bone mineral density and structure by menopause status, Seveso Women’s Health Study, Italy, 2008–2009.

Measurement		All women (*n *= 339)	Premenopause (*n *= 273)	Perimenopause/menopause (*n *= 66)^*a*^
Bone mineral density indices	
Bone mineral density (g/cm^2^)	Spine (L1–L4)	1.01 ± 0.13	1.02 ± 0.12	0.98 ± 0.14**
	Total hip	0.89 ± 0.12	0.90 ± 0.11	0.87 ± 0.14*
	Femoral neck	0.77 ± 0.11	0.78 ± 0.11	0.74 ± 0.12**
BMD *z*-score	Spine (L1–L4)	0.08 ± 1.13	0.10 ± 1.09	0.01 ± 1.30
	Total hip	–0.11 ± 0.98	–0.10 ± 0.94	–0.15 ± 1.14
	Femoral neck	–0.27 ± 0.98	–0.27 ± 0.94	–0.29 ± 1.11
Low BMD (*z*-score ≤ –1) [*n* (%)]	Spine (L1–L4)	61 (18.0)	46 (16.9)	15 (22.7)
	Total hip	64 (18.9)	46 (16.9)	18 (27.3)*
	Femoral neck	87 (25.7)	65 (23.8)	22 (33.3)
Bone strength indices	
Bending stress or cross sectional movement of inertia (cm^4^)	Narrow neck	2.27 ± 0.55	2.28 ± 0.55	2.25 ± 0.58
	Femoral shaft	3.11 ± 0.73	3.07 ± 0.70	3.27 ± 0.82**
	Intertrochanter	11.63 ± 2.70	11.53 ± 2.64	12.07 ± 2.94
Bending strength or section modulus (cm^3^)	Narrow neck	1.30 ± 0.27	1.31 ± 0.26	1.27 ± 0.31
	Femoral shaft	2.11 ± 0.38	2.09 ± 0.36	2.17 ± 0.43
	Intertrochanter	3.90 ± 0.77	3.87 ± 0.75	4.00 ± 0.85
Bone size	
Outer diameter (cm)	Narrow neck	3.17 ± 0.29	3.17 ± 0.30	3.20 ± 0.27
	Femoral shaft	2.85 ± 0.20	2.83 ± 0.20	2.91 ± 0.19**
	Intertrochanter	5.25 ± 0.30	5.23 ± 0.30	5.32 ± 0.30**
Cross sectional area (cm^2^)	Narrow neck	2.77 ± 0.44	2.78 ± 0.43	2.69 ± 0.50
	Femoral shaft	3.99 ± 0.60	3.98 ± 0.57	4.03 ± 0.72
	Intertrochanter	4.58 ± 0.76	4.59 ± 0.73	4.56 ± 0.85
Cortical thickness (cm)	Narrow neck	0.18 ± 0.03	0.18 ± 0.03	0.17 ± 0.03*
	Femoral shaft	0.56 ± 0.11	0.57 ± 0.11	0.55 ± 0.12
	Intertrochanter	0.39 ± 0.06	0.39 ± 0.06	0.38 ± 0.07*
Buckling ratio	Narrow neck	10.26 ± 2.83	10.08 ± 2.67	11.03 ± 3.34**
	Femoral shaft	2.70 ± 0.61	2.66 ± 0.57	2.85 ± 0.71**
	Intertrochanter	7.87 ± 1.46	7.78 ± 1.42	8.24 ± 1.60**
^***a***^*n* = 65 for HSA outcomes. Significantly different from the premenopause group at *p < 0.1, and **p < 0.05.				

Table 3 shows adjusted associations between a 10-fold increase in serum TCDD levels and spine, total hip, and hip neck BMD *z*-scores. We observed no significant associations (*p* < 0.05) between any of the measures of BMD with TCDD. All of the coefficients were positive, and were more strongly positive in the perimenopause/menopause group, but only the coefficient for bone size in the intertrochanter region showed a significant relationship at *p* < 0.05. No interactions with menopause status were significant (*p* > 0.2 for all measures). The odds of low BMD (*z*-score ≤ –1) at the spine or hip were also not associated with serum TCDD and there were no significant interactions by menopause status (see Supplemental Material, Table S2).

**Table 3:** Multivariable linear regression analyses for the relationship of serum TCDD (log_10_) with measures of bone mineral density and structure, Seveso Women’s Health Study, Italy, 2008–2009.

Measurement		Premenopause (*n *= 273) [Adj^*b*^ β (95% CI)]	Perimenopause/menopause (*n *= 66)^*a *^[Adj^*b*^ β (95% CI)]	*p*_int_
Bone mineral density indices	
Bone mineral density *Z*-score	Spine (L1–L4)	0.07 (–0.15, 0.29)	0.33 (–0.08, 0.74)	0.27
	Total hip	0.00 (–0.16, 0.17)	0.19 (–0.12, 0.49)	0.28
	Femoral neck	0.04 (–0.13, 0.21)	0.23 (–0.10, 0.55)	0.31
Bone strength indices	
Bending stress or cross sectional moment of inertia (cm^4^)	Narrow neck	0.09 (–0.00, 0.18)*	0.10 (–0.08, 0.27)	0.95
	Femoral shaft	0.07 (–0.03, 0.18)	0.17 (–0.03, 0.37)*	0.38
	Intertrochanter	0.42 (–0.01, 0.85)*	0.29 (–0.51, 1.10)	0.78
Bending strength or section modulus (cm^3^)	Narrow neck	0.05 (–0.00, 0.09)*	0.05 (–0.04, 0.13)	1.00
	Femoral shaft	0.04 (–0.01, 0.09)	0.08 (–0.02, 0.18)*	0.42
	Intertrochanter	0.08 (–0.05, 0.20)	0.10 (–0.14, 0.33)	0.90
Bone size	
Outer diameter (cm)	Narrow neck	0.02 (–0.03, 0.08)	0.00 (–0.10, 0.11)	0.74
	Femoral shaft	0.01 (–0.02, 0.05)	0.03 (–0.03, 0.10)	0.59
	Intertrochanter	0.06 (0.01, 0.11)**	–0.00 (–0.10, 0.09)	0.25
Cross sectional area (cm^2^)	Narrow neck	0.03 (–0.04, 0.10)	0.07 (–0.06, 0.20)	0.65
	Femoral shaft	0.06 (–0.03, 0.15)	0.11 (–0.05, 0.28)	0.60
	Intertrochanter	0.06 (–0.07, 0.19)	0.08 (–0.17, 0.32)	0.93
Cortical thickness (cm)	Narrow neck	0.00 (–0.00, 0.01)	0.00 (–0.01, 0.01)	0.65
	Femoral shaft	0.01 (–0.01, 0.02)	0.01 (–0.03, 0.04)	0.87
	Intertrochanter	0.00 (–0.01, 0.02)	0.00 (–0.02, 0.02)	0.91
Buckling ratio	Narrow neck	0.08 (–0.47, 0.63)	–0.30 (–1.33, 0.74)	0.52
	Femoral shaft	–0.01 (–0.13, 0.10)	0.01 (–0.20, 0.22)	0.85
	Intertrochanter	0.06 (–0.22, 0.33)	0.01 (–0.51, 0.53)	0.88
Abbreviations: Adj, adjusted; *p*_int_, *p*_interaction_. ^***a***^*n* = 65 for HSA outcomes. ^***b***^BMD models adjusted for age at explosion and BMI. HSA ­models adjusted for age at explosion, height, and weight. On coefficient *p < 0.1, and **p < 0.05.				

As presented in Table 4, within the premenopause group, we found no evidence of effect modification by exposure before or after age of peak bone mass. However, there was an interaction by exposure at 5 years of age, with those exposed earlier showing a positive association for TCDD and BMD *z*-score at the femoral neck (*n* = 46, adjusted β = 0.29; 95% CI: –0.03, 0.62) versus a negative association if exposed after (*n* = 227, adjusted β = –0.08; 95% CI: –0.28, 0.12) (*p*_interaction_ = 0.06) (Table 5). In contrast, within the perimenopause/menopause group, an increase in TCDD levels was negatively associated with hip (total, neck) BMD *z*-score in those exposed before age at peak bone mass (*n* = 18, adjusted β = –0.16; 95% CI: –0.85, 0.53 for total hip, adjusted β = –0.24; 95% CI: –1.00, 0.51 for femoral neck) but positively associated with those exposed after (*n* = 48, adjusted β = 0.39; 95% CI: –0.01, 0.78 for total hip, adjusted β = 0.50; 95% CI: 0.07, 0.93 for femoral neck) (*p*_interaction_ = 0.09 for neck and 0.17 for total) (Table 4).

**Table 4:** Multivariable linear regression analyses for the relationship of serum TCDD (log_10_) with measures of bone mineral density and structure, stratified by menopause status and exposure before or after age at peak bone mass, SWHS, 2008–2009.

Measurement		Premenopause		*p*_int_	Perimenopause/menopause		*p*_int_
		Exposure before peak bone mass (*n *= 219) [Adj^*b*^ β (95% CI)]	Exposure after peak bone mass (*n *= 54) [Adj^*b*^ β (95% CI)]		Exposure before peak bone mass (*n *= 18) [Adj^*b*^ β (95% CI)]	Exposure after peak bone mass (*n *= 48)^*a *^[Adj^*b*^ β (95% CI)]	
Bone mineral density indices	
Bone mineral density *z*‑score	Spine (L1–L4)	0.03 (–0.21, 0.26)	0.20 (–0.36, 0.75)	0.58	0.15 (–0.87, 1.17)	0.47 (–0.12, 1.05)	0.59
	Total hip	–0.02 (–0.19, 0.16)	0.03 (–0.39, 0.45)	0.84	–0.16 (–0.85, 0.53)	0.39 (–0.01, 0.78)*	0.17
	Femoral neck	0.01 (–0.18, 0.20)	0.11 (–0.34, 0.55)	0.69	–0.24 (–1.00, 0.51)	0.50 (0.07, 0.93)**	0.09
Bone strength indices	
Bending stress or cross sectional moment of inertia (cm^4^)	Narrow neck	0.12 (0.02, 0.22)**	0.01 (–0.23, 0.25)	0.40	–0.22 (–0.65, 0.21)	0.18 (–0.05, 0.41)	0.09
	Femoral shaft	0.13 (0.01, 0.24)**	–0.15 (–0.43, 0.12)	0.07	–0.14 (–0.64, 0.35)	0.33 (0.07, 0.59)**	0.08
	Intertrochanter	0.55 (0.08, 1.03)**	0.03 (–1.08, 1.15)	0.40	–0.77 (–2.87, 1.32)	0.58 (–0.53, 1.68)	0.23
Bending strength or section modulus (cm^3^)	Narrow neck	0.06 (0.01, 0.11)**	–0.01 (–0.13, 0.11)	0.25	–0.10 (–0.34, 0.15)	0.10 (–0.03, 0.24)	0.14
	Femoral shaft	0.06 (0.01, 0.12)**	–0.06 (–0.20, 0.07)	0.09	–0.11 (–0.36, 0.14)	0.18 (0.05, 0.31)**	0.03
	Intertrochanter	0.11 (–0.03, 0.24)	0.01 (–0.32, 0.34)	0.60	–0.21 (–0.81, 0.40)	0.18 (–0.14, 0.50)	0.24
Bone size	
Outer diameter (cm)	Narrow neck	0.02 (–0.04, 0.09)	0.07 (–0.08, 0.22)	0.54	–0.16 (–0.39, 0.08)	–0.03 (–0.16, 0.09)	0.34
	Femoral shaft	0.03 (–0.01, 0.07)	–0.06 (–0.15, 0.03)	0.08	0.02 (–0.12, 0.17)	0.06 (–0.02, 0.14)	0.65
	Intertrochanter	0.07 (0.01, 0.12)**	0.02 (–0.11, 0.15)	0.51	–0.13 (–0.36, 0.09)	–0.02 (–0.14, 0.10)	0.34
Cross sectional area (cm^2^)	Narrow neck	0.05 (–0.03, 0.12)	–0.00 (–0.18, 0.18)	0.61	–0.26 (–0.62, 0.09)	0.18 (–0.00, 0.37)*	0.02
	Femoral shaft	0.08 (–0.01, 0.18)*	–0.01 (–0.24, 0.21)	0.44	–0.24 (–0.66, 0.17)	0.24 (0.02, 0.46)**	0.03
	Intertrochanter	0.09 (–0.05, 0.23)	–0.02 (–0.35, 0.31)	0.54	–0.28 (–0.88, 0.33)	0.23 (–0.08, 0.55)	0.12
Cortical thickness (cm)	Narrow neck	0.00 (–0.00, 0.01)	–0.00 (–0.02, 0.01)	0.62	–0.01 (–0.04, 0.02)	0.01 (–0.00, 0.03)*	0.13
	Femoral shaft	0.00 (–0.02, 0.02)	0.02 (–0.03, 0.07)	0.58	–0.06 (–0.14, 0.02)	0.02 (–0.02, 0.07)	0.07
	Intertrochanter	0.00 (–0.01, 0.02)	0.01 (–0.03, 0.04)	0.84	–0.03 (–0.08, 0.03)	0.01 (–0.02, 0.04)	0.15
Buckling ratio	Narrow neck	–0.03 (–0.61, 0.56)	0.84 (–0.53, 2.21)	0.26	–0.05 (–3.13, 3.02)	–1.10 (–2.72, 0.52)	0.52
	Femoral shaft	–0.00 (–0.12, 0.12)	–0.13 (–0.42, 0.16)	0.42	0.23 (–0.34, 0.81)	–0.04 (–0.35, 0.26)	0.37
	Intertrochanter	0.07 (–0.23, 0.38)	–0.12 (–0.84, 0.60)	0.62	0.35 (–1.02, 1.71)	–0.10 (–0.82, 0.62)	0.54
Abbreviations: Adj, adjusted; *p*_int_, *p*_interaction_. ^***a***^*n *= 47 for HSA outcomes. ^***b***^BMD models adjusted for age at explosion and BMI. HSA models adjusted for age at explosion, height, weight, age began athletic training, and hours per week of exercise. On coefficient *p < 0.1, and **p < 0.05.							

**Table 5:** Multivariable linear regression analyses for the relationship of serum TCDD (log_10_) with measures of bone mineral density and structure in premenopausal women, stratified by age at explosion (< 5 and ≥ 5 years), Seveso Women’s Health Study, Italy 2008–2009.

Measurement		Age < 5 years at explosion (*n *= 46) [Adj^*a*^ β (95% CI)]	Age ≥ 5 years at explosion (*n *= 227) [Adj^*a*^ β (95% CI)]	*p*_int_
Bone mineral density indices	
Bone mineral density *z*-score	Spine (L1–L4)	0.15 (–0.26, 0.56)	0.03 (–0.23, 0.28)	0.62
	Total hip	0.17 (–0.14, 0.48)	–0.07 (–0.27, 0.12)	0.19
	Femoral neck	0.29 (–0.03, 0.62)*	–0.08 (–0.28, 0.12)	0.06
Bone strength indices	
Bending stress or cross sectional moment of inertia (cm^4^)	Narrow neck	0.15 (–0.03, 0.33)	0.09 (–0.02, 0.20)	0.58
	Femoral shaft	0.27 (0.07, 0.47)**	0.01 (–0.11, 0.14)	0.04
	Intertrochanter	0.60 (–0.23, 1.43)	0.43 (–0.08, 0.94)*	0.74
Bending strength or section modulus (cm^3^)	Narrow neck	0.11 (0.02, 0.20)**	0.03 (–0.02, 0.09)	0.16
	Femoral shaft	0.14 (0.04, 0.25)**	0.01 (–0.05, 0.07)	0.03
	Intertrochanter	0.14 (–0.11, 0.38)	0.08 (–0.07, 0.23)	0.68
Bone size	
Outer diameter (cm)	Narrow neck	–0.02 (–0.13, 0.10)	0.05 (–0.02, 0.12)	0.33
	Femoral shaft	0.05 (–0.02, 0.12)	0.00 (–0.04, 0.04)	0.21
	Intertrochanter	0.04 (–0.05, 0.14)	0.07 (0.01, 0.13)**	0.64
Cross sectional area (cm^2^)	Narrow neck	0.16 (0.03, 0.29)**	–0.00 (–0.08, 0.08)	0.04
	Femoral shaft	0.23 (0.06, 0.40)**	0.01 (–0.09, 0.11)	0.03
	Intertrochanter	0.21 (–0.04, 0.46)*	0.03 (–0.13, 0.18)	0.22
Cortical thickness (cm)	Narrow neck	0.01 (0.00, 0.02)**	–0.00 (–0.01, 0.00)	0.03
	Femoral shaft	0.02 (–0.02, 0.06)	–0.00 (–0.02, 0.02)	0.31
	Intertrochanter	0.01 (–0.01, 0.03)	0.00 (–0.01, 0.02)	0.54
Buckling ratio	Narrow neck	–0.77 (–1.79, 0.25)	0.42 (–0.21, 1.05)	0.05
	Femoral shaft	–0.05 (–0.26, 0.16)	–0.01 (–0.15, 0.12)	0.77
	Intertrochanter	–0.11 (–0.64, 0.43)	0.09 (–0.24, 0.42)	0.54
Abbreviations: Adj, adjusted; *p*_int_, *p*_interaction_. ^***a***^BMD models adjusted for age at explosion and BMI. HSA models adjusted for age at explosion, height, weight, age began athletic training, and hours per week of exercise. On coefficient *p < 0.1, and **p < 0.05.				

Table 3 shows that a 10-fold increase in TCDD was positively and in some cases, significantly, associated with measures of bone strength and size; these relationships did not differ significantly by menopause status (*p* < 0.2). However, as shown in Table 4, this relationship differed somewhat by whether TCDD exposure occurred before or after age at peak bone mass. For example, in perimenopausal/menopausal women who were exposed after peak bone mass (*n* = 48), higher TCDD was associated with greater bone strength, but in those exposed before peak bone mass (*n* = 18), higher TCDD levels were largely associated with lower bone strength. This interaction was statistically significant (*p* < 0.2) for bone strength of the narrow neck and femoral shaft; sample sizes, however, were small. In contrast, for premenopausal women who were exposed after peak bone mass (*n* = 54), there was no clear relationship between TCDD and any of the structural measures, but in those exposed before peak bone mass (*n* = 219), log_10_TCDD was positively related to some measures of bone strength (narrow neck and femoral shaft) and size (intertrochanter). As shown in Table 5, the coefficients between TCDD and bone strength become somewhat stronger for those premenopausal women who were < 5 years of age at the explosion. For example, a 10-fold increase in TCDD was associated with an increase of 0.27 cm^4^ (95% CI: 0.07, 0.47) in femoral shaft bending strength in women who were < 5 years of age before the explosion, but showed little association with those who were ≥ 5 years of age (β = 0.01; 95% CI: –0.11, 0.14; *p*_interaction_ = 0.04) We also observed similar interactions at the femoral shaft (bending strength and cross-sectional area) and the narrow neck (cross-sectional area, cortical thickness, and buckling ratio), with the younger group showing associations of greater magnitude and statistical significance.

## Discussion

This is the first study to examine the relationship of serum TCDD and bone health in women. We examined this association in a population with relatively high exposure to TCDD due to an industrial explosion in Seveso, Italy, in 1976. We found little evidence that exposure to TCDD had an adverse relationship with bone health > 30 years later; in fact, some trends suggested better bone density. In addition, we observed evidence of better bone structure associated with TCDD levels in premenopausal women who were exposed before peak bone mass and an even stronger association in those who were exposed before the onset of puberty (< 5 years of age) although the sample size was small (*n* = 46). In contrast, among postmenopausal women, exposure after the age of peak bone mass was associated with evidence of better bone structure, though estimates should be interpreted with caution given the small sample size.

Few studies in humans have examined the relation of dioxin-like compounds and bone health. In a case series, Miller (1985) reported that 3 of 12 Japanese children exposed *in utero* to PCB-contaminated rice oil including dioxin-like furans (Yusho) had natal teeth, open fontanelles, and unusual calcification of the skull at birth, which he suggested might have been attributable to a perturbation in calcium metabolism. In a similar accident in Taiwan (Yucheng), natal teeth and open fontanelles were also noted in 11 of 127 children at birth (Rogan et al. 1988), but there were no observed associations with BMD measured at 9 years of age (*n* = 25 exposed and 25 controls) (Guo et al. 1994). In 153 Inuit menopausal women, serum levels of dioxin-like PCB-105 or 118 were not associated with osteoporosis-related ultrasound measurements of the calcaneum (Cote et al. 2006). An ecologic study of Swedish east coast fishermen and wives who consume fish from the Baltic Sea (known to be contaminated with persistent organochlorine compounds including dioxin-like compounds) had a higher incidence rate of vertebral fractures but not hip fractures compared with west coast fishermen and wives who were presumed to consume fish with lower exposure (Alveblom et al. 2003). In a follow-up study of 115 Swedish men, serum levels of some dioxin-like PCBs were significantly positively associated with BMD (PCB-167), while others were not (PCBs 105, 118, 156) (Glynn et al. 2000). In a study of older (60–81 years) Swedish males (*n* = 154) and females (*n* = 167) living near the Baltic coast, serum measures of dioxin-like PCB-118 were negatively associated with BMD of the forearm in males, but positively associated with BMD in females (Hodgson et al. 2008).

One limitation of the present study is that because of the low volume of serum sample, only TCDD and not the full toxic equivalency (TEQ) constituents could be measured in archived specimens. A previous analysis of pooled blood specimens of residents in the unexposed zone (non-ABR) indicated that analytes other than TCDD contributed approximately 80 ppt, on average, to the total TEQ (Eskenazi et al. 2004), and results were similar when exposures were defined using new toxic equivalency factor values published in 2005 (Warner et al. 2013). If these background levels are similar in all zones, individuals with low levels of TCDD might still have substantial total TEQ levels. Because we considered only TCDD, we may have underestimated the health effects of exposures to dioxin-like compounds experienced by Seveso women. Nevertheless, in the only previous study on tooth or bone development in the Seveso cohort, maternal TCDD serum levels were associated with developmental enamel defects, with almost all defects occurring in those < 5 years at the time of the explosion (Alaluusua et al. 1999, 2004).

We are not aware of previous epidemiologic studies that have specifically examined the relation of TCDD and bone health, but several studies in rats have reported evidence of effects, particularly when exposure occurs during early development (Alaluusua and Lukinmaa 2006; Jamsa et al. 2001; Miettinen et al. 2005). Although both adult and *in utero/*lactational TCDD exposure was shown to alter bone geometry and decrease mechanical strength in several different rat strains (Finnila et al. 2010; Jamsa et al. 2001; Miettinen et al. 2005), only early-life exposure was associated with changes in bone mineral density (Finnila et al. 2010; Miettinen et al. 2005). Interestingly, a follow-up study showed the observed negative effects of *in utero* TCDD exposure on bone density, bending force, and mineralization were reversed at the age of 1 year (Miettinen et al. 2005). Bone is constantly modeled and remodeled, so bone may have the ability to recover from an insult with time. In the SWHS, we could not evaluate associations with *in utero* exposures, because all women were exposed postnatally. Also, we measured bone density > 30 years after the explosion, and thus would not have observed temporary changes in bone soon after exposure if they occurred.

Although we have included in the present investigation only the group whom we hypothesized to be the most susceptible subpopulation (women who were young at exposure), we have not followed most of these women to the age when they would be most at risk for poorer bone health—that is, after menopause. Ideally, we would follow these women to record increased risk for bone fracture, because the measures obtained by DXA are only surrogate markers of bone fragility (Kanis 2007). In addition, future studies should include biomarker measures of bone health (Kanis 2007).

In addition, it is likely that not all humans are equally susceptible to the effects of TCDD, given that there was a 10- to 49-fold difference in TCDD toxicity among rat strains, attributed to genetic differences in the AhR (Herlin et al. 2010); and in the more resistant strains, effects were seen only at higher doses (Jamsa et al. 2001). Evidence supporting an effect of estrogen status on the bone response is provided by studies showing increased BMD in estrogen-deprived ovariectomized adult rats exposed to dioxin-like PCB-126 (Lind et al. 1999, 2000, 2004). Nevertheless, in the SWHS, we did not observe large or statistically reliable differences in associations between TCDD and BMD or bone structure by menopausal status; however, all women were premenopausal at the time of TCDD exposure.

Some of our observed findings may be attributed to dioxin’s more pronounced inhibitory effect on ER-β, a negative modulator of periosteal bone formation, than on ER-α, which enhances bone formation, resulting in an imbalance in the ratio of ER-β/ER-α activity (Rüegg et al. 2008; Saxon et al. 2007). This imbalance might result in dioxin-associated increased bone strength and size among certain groups of women.

The lack of adverse association in the present study is not likely attributable to dose. The equivalent human body burden for the no observable adverse effect level for effects of TCDD in the most sensitive rats on bone growth, modeling, and mechanical strength is estimated to be approximately 11 ng/kg body burden (Jamsa et al. 2001). The median lipid-adjusted serum TCDD level (73 ppt or ~ 15 ng/kg body burden) measured in this sample of Seveso women is somewhat higher.

This study did have important strengths: It is the largest to date on exposure to dioxin-like compounds and bone health in women. Unlike previous epidemiologic studies of dioxin-like compounds, we measured TCDD in serum collected near the time of explosion, and we used state-of-the-art methods to measure bone density as well as structure.

In summary, we did not find evidence of long-term adverse effects of postnatal exposure to TCDD in a cohort of women heavily exposed in Seveso, Italy. In fact, in some cases, we observed improved bone measures associated with TCDD exposure. However, we did not include the segment of the population whom we believe to be most susceptible to adverse effects—those exposed *in utero*. We plan to follow this group, the children of the SWHS cohort, in future studies. In addition, we plan to follow the women who were young at the time of exposure (< 5 years) and still premenopausal at last follow-up to determine whether early-childhood TCDD exposure affects longer-term bone health. In addition to dose, timing of exposure, and estrogen status, future epidemiologic studies should consider potential genetic markers of susceptibility.

## Supplemental Material

(172 KB) PDF
