# Supplementary material for: Serum Dioxin Concentrations and Bone Density and Structure in the Seveso Women’s Health Study
Source: Environ Health Perspect. 2013 Nov 15;122(1):51–7. doi: 10.1289/ehp.1306788 (PMC3888571; doi:10.1289/ehp.1306788)
Supplement: (172 KB) PDF [file ehp.1306788.s001.508.pdf]

Environmental Health Perspectives doi:10.1289/ehp.1306788

Supplemental Material

Serum Dioxin Concentrations and Bone Density and Structure in the Seveso Women's Health Study

Brenda Eskenazi, Marcella Warner, Marcella Sirtori, Thomas Fuerst, Stephen A. Rauch, Paolo Brambilla, Paolo Mocarelli, and Alessandro Rubinacci

Supplemental Material, Table S1. Distribution of serum TCDD concentrations measured in serum collected near the time of the 1976 explosion by menopause status, Seveso Women's Health Study, Italy 2008-2009.

Sample	n	Geometric Mean ± GSD	Min	Percentiles 25th	50th	75th	Max
All women	340	86.7 ± 4.2	3.9	33.1	73.2	193.0	56,000
Premenopause	274	95.2 ± 4.2	3.9	40.9	78.9	209.0	56,000
Perimenopause/menopause	66	58.8 ± 4.4	3.9	21.9	43.1	129.0	2,820

GSD = geometric standard deviation.

Supplemental Material, Table S2. Multivariable logistic regression analyses for the relationship of serum TCDD (log_{10}) with low BMD (Z-score ≤ -1) at the spine and hip, by menopause status, Seveso Women's Health Study, Italy 2008-2009.

	Premenopause (n = 273)		Perimenopause/menopause (n = 66)		
**Measurement**	**Cases/Total**	**Adj.[a] OR (95% CI)**	**Cases/Total**	**Adj.[a] OR (95% CI)**	**p-*int***
Spine (L1-L4)	46/273	1.24 (0.72, 2.13)	15/66	0.61 (0.21, 1.80)	0.25
Total hip	46/273	1.00 (0.55, 1.84)	18/66	1.15 (0.40, 3.33)	0.83
Femoral neck	65/273	0.97 (0.57, 1.64)	22/66	0.98 (0.37, 2.61)	0.99

[a]Adjusted for age at explosion and BMI.
